# The LINKIN Health Census process: design and implementation

**DOI:** 10.1186/1472-6963-12-321

**Published:** 2012-09-18

**Authors:** Catherine Elizabeth Hoon-Leahy, Jonathan Newbury, Alison Kitson, Deirdre Whitford, Anne Wilson, Jonathan Karnon, Jenny Baker, Konrad Jamrozik, Justin Beilby

**Affiliations:** 1 School of Population Health, University of Adelaide, Adelaide, Australia; 2 School of Nursing, University of Adelaide, Adelaide, Australia; 3 Faculty of Health Sciences, University of Adelaide, Adelaide, Australia

## Abstract

This paper describes the first phase of the LINKIN Health Study, which aims to evaluate health system functioning within a rural population. Locally relevant data on the health status and service usage of this population, including non-users and users, health service providers traditionally omitted from health services research, and multiple socio-economic indicators, was collected using a self-complete health census. Household response was 75% (N = 4425). Response was greater when face-to-face contact was made at delivery compared to when questionnaires were left in the letterbox (89% vs 64%), falling to 26% when no face-to-face contact was made at either delivery or collection.

## Background

The LINKIN Health Study aims to examine and influence health system effectiveness on a defined population the rural centre of Port Lincoln, South Australia, as a case study. This study focuses on health status and patterns of use/non-use of a full range of health services for this specific population, with the intention of informing policy reform and practical health interventions. The need for comprehensive, locally relevant health data is recognised as a key requirement for effective monitoring and evaluation of healthcare delivery at a local level [1,2]. Creating new models of health must be built on the foundations of accurate, locally detailed health data. Gaps remain in generating detailed, reliable population health data beyond national, standard sources, leaving complexities of health needs and health service utilisation within a local health system context poorly understood. The LINKIN Health Study overall approach connects a baseline population health census to in-depth analyses of health service usage experiences and preferences for care within different sub-populations, while translating the emerging set of knowledge into locally appropriate opportunities for health service redesign through iterative stakeholder engagement. In line with contemporary concerns with translating knowledge into application, the LINKIN Health Study design will then aim to generate practical and locally relevant evidence and measures of progress, incorporating participatory implementation processes [2].

Whilst transactional data (electronic data collected through service interactions) has potential to be effectively used [3-6] for health system research, within this South Australian context, there are short term legal and ethical barriers to the efficient implementation of a person-based data linkage approach [7-9]. The LINKIN Health Study purposefully took a broad approach to defining health services and thus a different approach had to be adopted to include health services where transactional data may not be available, e.g. Aboriginal health services, chiropractors, social workers and naturopaths. Moreover, a priority to gather data from both users and non-users of services, and the need to examine health and service usage in relation to person-based socio-economic position – not necessarily recorded with transactional data – confirmed the need for an alternative approach.

The first phase of the LINKIN Health Study was a voluntary health census of all residents living in the Local Government Area (LGA) of Port Lincoln. The health census was conducted over a six week period from September to November 2010 and included all residents 15 years of age and over, excluding those who spent the entire census period in hospital or in the dementia units of the two local nursing homes.

The census was essential in providing: i) comprehensive set of data from local residents, rather than the less reliable data obtained from a targeted survey, ii) detailed baseline health and socio-economic data from individual respondents, iii) a broad view of all health services, including alternative and complementary medicine, iv) a community perspective of health and the way the whole population within this community used the health services, including the views of those not using services, and v) a sample of the population who agreed to be contacted for further detailed studies following the census.

Port Lincoln is a remote city on the Eyre Peninsula in South Australia with a population of approximately 14000 people and approximately 5000 private dwellings [10]. It is 650 kilometres by road from the closest major centre, Adelaide (population 1.2 million) [11], the capital city of South Australia. Approximately 5% of the population in Port Lincoln are Aboriginal people. The University of Adelaide has infrastructure in Port Lincoln as part of its rural medical education program.

This paper outlines the design and implementation process of the LINKIN health census. A census is differentiated from survey techniques as it aims to have complete enumeration of a community rather than a proportion of a population.

## Methods

### Questionnaire development

Two questionnaires were designed, one to gather information about the household (the census household questionnaire, CHQ; with a slightly modified CHQ designed for non-private households) and another to gather information about each eligible individual living in the household (the census individual questionnaire, CIQ). The questions covered four key areas: social determinants of health, health status (self-rated health and attitude to prevention), health conditions (self-reported), and health service usage and any difficulties accessing services. Social determinant data included age, gender, ethnicity, educational attainment, housing, employment, health insurance cover and tobacco usage.

Given the broad target population, including people with varied literacy levels, and the chosen self-complete delivery mode, the research team considered it paramount that the CIQ was no longer than 4 x A4 pages, printed on a double-sided, folded A3 page so that only one piece of paper was given to an individual. The CHQ was a separate one page A4 questionnaire and only one of these was delivered per household. Particular care was taken to give the questionnaires a professional aesthetically pleasing look with as much white space as was possible. Specific effort was given to encouraging response from hard to reach groups, through use of plain direct questions, the development of an audio/visual support DVD provided on request, and the use of interviewers to assist in completion of the questionnaire if requested. As with all material related to the census and the broader study, questionnaires were branded with the LINKIN Health Study logo. Selected graphics and colour palette were defined to create a uniform appearance for the promotional and census material delivered to the population across various media. The CIQ and CHQ were pilot tested by an independent researcher using Cognitive Interview Testing and Test Retest techniques [12]. Minor modifications were made as a consequence of these piloting activities.

### Census operation

The census component of the LINKIN Health Study was approved by the University of Adelaide’s Human Research Committee (H-036-2010). The health census was scheduled to occur over a period of six weeks, with three weeks for delivery and three weeks for collection. A nominal date for completing the census was chosen (10 October, 2010); thus 10.10.10, a recognisable date to promote and remember, occurred in the middle of the census period and formed an effective advertising and marketing slogan. The health census was hand-delivered to every household within the Port Lincoln LGA. The LGA has 26 Census Districts (CD) as defined by the Australian Bureau of Statistics’ Census. A minimum of three attempts to deliver and three attempts to collect the census questionnaires was made at each household with self-addressed envelopes left for postal return if face-to-face contact had not been established.

### Personnel

The LINKIN Health Study team consisted of eight investigators and two research fellows. A Community Engagement Officer was employed to promote and engage the community in the health census. A Census Supervisor (CS) was recruited to take an overview of the process and manage the staff in the field. Four Team Leaders (TLs) were recruited, each to manage seven Census Collectors (CCs) with each CC assigned to one CD. Two Data Administrators (DAs) were employed to enter the field management data which was generated by the CCs.

**Figure 1:**
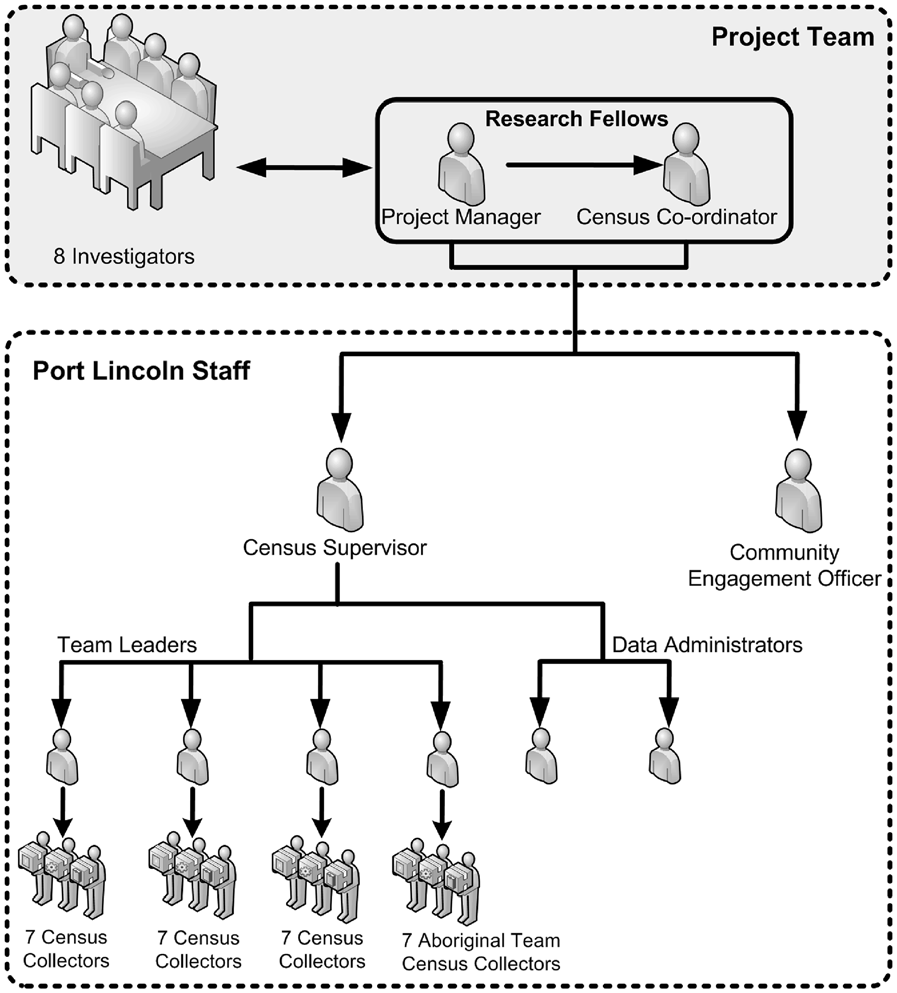
Health Census Personnel for the LINKIN Health Census, Port Lincoln, Australia, 2010.

A specific strategy to provide a culturally appropriate approach to data collection for the Port Lincoln Aboriginal people involved the development of an Aboriginal team of CCs led by an Aboriginal TL to work in districts identified as having a high proportion of Aboriginal residents. In this team of seven CCs, five were Aboriginal. In two of this team’s CDs, where there were a higher proportion of low socio-economic households, a pair of CCs worked together. Thus a total of thirty-six staff members from Port Lincoln were recruited to conduct the census component (see Figure 1).

### Development of census procedures

The structure and design of the census took an initial focus on how the CC would perform the tasks of delivery and collection of the census questionnaires in the field, including all materials, instructions, training and supervision required. In summary, the CCs had to perform the following tasks:

•Identify each household within their district

• Visit each household and attempt to make contact with a householder in order to inform them of the voluntary health census

• Record each visit to the household and whether or not contact was made

•Record the number of CIQs and CHQs delivered to each household

• Record details about any refusals by the householders

• record any relevant comments about the household (e.g. problems with access, aggressive dog, etc.).

**Figure 2:**
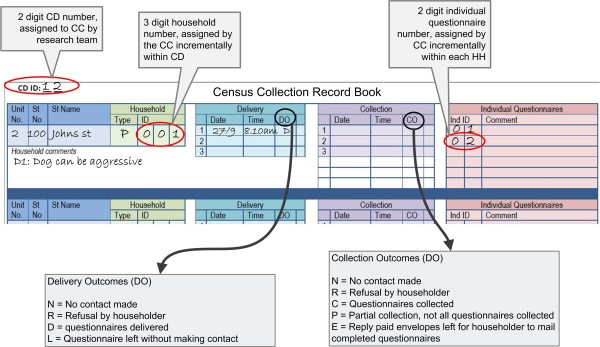
Health Census Collection Record Book with Delivery/Collection Outcome Codes Defined.

The CCs were also required to provide enough information for management to track the progress of the field work. The field data was collected via the Census Collection Record Book (CCRB) (see Figure 2). This document was pivotal in driving the census by providing data for the computerised Field Management System (FMS) which was in turn used by the management team (TLs, CS and research fellows) to determine delivery and collection progress across CDs.

The CC generated a unique identification number (ID) for the questionnaires based on CD number, household number and a number assigned to each eligible individual within the household, for example in Figure 2, the unique ID would be 12/001/02. This unique ID was recorded on the CCRB and then transferred to the questionnaires. As Figure 2 shows, a CCRB entry also included other information, e.g. address, outcome codes for each delivery attempt (DO) and collection attempt (CO).

**Figure 3:**
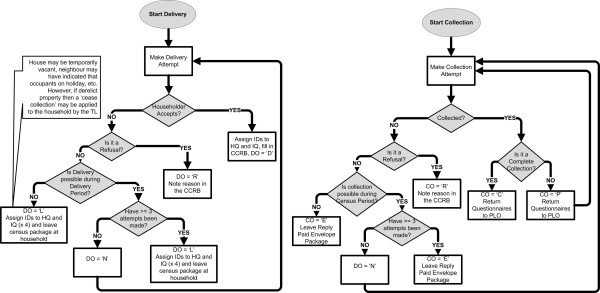
Delivery/Collection Attempt Procedures for the LINKIN Health Census: Indicating Delivery and Collection Outcome Codes.

Field procedures were systematically identified by the research fellows using scenario role plays from the perspective of householder, CCs, TLs and DAs. As a consequence of working through these role plays decision matrixes were generated to cover all interaction possibilities for tracking census delivery and collection progress. Summaries of the delivery and collection procedures are outlined in Figure 3. The scenario role plays were important in identifying equipment required by the CCs, TLs and DAs (refer to Additional file 1: Table S1). In addition, issues relating to occupational health and safety and confidentiality were also clearly outlined during the role plays. Further, the role plays highlighted the importance of the initial communication with the householder to maximise response rate success. All the knowledge gathered from the role plays was reflected in step-by-step procedures documented in the LINKIN Census Staff Manual.

Occupational health and safety issues were paramount for the CCs in the field. The University and the labour hire agency provided advice, training and appropriate working arrangements to address issues tied to personal safety, e.g. ID card, emergency telephone contacts, personal alarms, training on how to deal with difficult interactions with people and animals, working hour arrangements, and lifting and carrying census materials. Privacy and confidentiality of material, including questionnaire data, was ensured through a variety of measures such as the use of confidentiality agreements for all staff, police checks for all those employed on the census, the use of privacy envelopes for all returning questionnaires, the storage of all field material in locked cabinets and the use of restricted login access on the computerised FMS.

### Field management system

The FMS was a computerised system for storing the census process data reported by the CC via the CCRB. The FMS incorporated data entry screens, search screens and reports so that the research fellows and census management staff could i) track the progress of the CCs in the field, ii) make decisions on how best to manage census delivery and collection, and iii) obtain quick and timely summaries of data collection progress (see Table 1). The FMS was a critical component in providing the data for the research fellows to calculate accurate response rates throughout the fieldwork phase.

**Table 1:** Components of computerised field management system for the LINKIN Health Census, Port Lincoln, Australia, 2010

	
**Main Menu Screen**	Allow navigation to other components of the FMS. Used by Census Co-ordinator, TLs, DAs, CS and Project Manager.
**CCRB Entry Screen**	To allow the entry of household data from the CCRB. This screen also recorded delivery and collection attempts, the IDs of the CIQs delivered and any comments. Only the DAs entered CCRB data. TLs and the Census Co-ordinator could also make comments in the comments area and indicate when collection attempts were to cease on a particular household (e.g. in the case of derelict properties).
**Household Delivery Search Screen**	This screen allowed search functions on CD number, household number, household address or a specified delivery outcome. This screen was used primarily by the TLs to track households in a specified CD. For example whether all streets in the CD had been visited, etc. Also very useful for tracking duplication of households in the CCRB or duplication of household numbers within a CD.
**Household Collection Search**	This allowed search functions on CD number, household number, household address or a specified collection outcome. Used by TLs, to track collection progress within a CD.
**Questionnaires Received Entry Screen**	This screen recorded the IDs of questionnaires that were collected. A report was generated for a specified day listing all questionnaires entered on that day. The questionnaires and report were then sent to Adelaide for data entry. Used by the DAs.
**Staff Screen**	A list of all the staff with their contact details for quick access.
**Delivery Attempts by Delivery Outcome Report / Collection Attempts by Collection Outcome Report**	Summary of the delivery or collection outcomes for each delivery/collection attempt for each CD. Used by Census Co-ordinator, Project Manager and CS to monitor delivery/collection progress across all CDs. TLs also used this to compare progress amongst their CDs.
**Delivery Follow Up Report/ Collection Follow Up Report**	Lists households that may require follow up during the delivery or collection phase (where contact had not yet been made or the questionnaires were left prematurely or partially collected). Used by TLs.
**Final Delivery Outcomes Report / Final Collection Outcomes Report**	Summary of the final delivery or collection outcomes for each CD including percentages. This was an additional report requested by the TLs and CS. It was originally thought best not to provide such a report as it would focus TLs on comparisons when CDs are inherently different.
**Household Comments Report**	List all households for specified CD where comments have been made. Used to identify where CCs may be experiencing some difficulties. Used by the TLs.
**Individual Participant Comments Report**	Lists all households for a specified CD where an individual has requested assistance in completing the questionnaire. TLs used this report to determine which households they needed to visit in order to conduct an interview.
**Household Summary Report**	Lists all households for a specified CD, indicating delivery and collection outcomes, number of CIQs delivered/collected, cessation of collections and all comments. Used by Census Co-ordinator, Project Manager and TLs. This report was written in the field when it became apparent that a summary document was required.
**Questionnaires Entered Log**	Lists the IDs of all CHQs and CIQs that were entered into the FMS on a specified day. Used by DAs, CS and the Adelaide Data Entry organisation.

The FMS was developed as a Microsoft Access database with a backend and frontend configuration. The FMS ran on a standalone peer-to-peer PC network consisting of six PCs, with one PC acting as a server and running the backend. The system was isolated from the internet for two reasons: firstly to guarantee security of the process data, which included the addresses of all households in the area, and secondly, as the internet was known to be patchy at the site it was decided not to risk delays in accessing the system remotely. Security software was installed to prevent any downloads or access to any devices plugged into the system. Regular backups were conducted throughout the working day and copies stored offsite. The Census Co-ordinator was the only person holding the password to bypass the security software.

### Infrastructure requirements

Several rooms were made available in the University’s medical education building centrally based in Port Lincoln. The largest room housed desk space for the four TLs, the two DAs and the visiting research fellows. The Port Lincoln Office (PLO) was the hub of census activity with CCs regularly visiting the PLO to report to their TLs.

The PLO included electronic equipment and secure storage facilities. The CS, TLs and DAs were provided with computers and printer access in order to use the computerised FMS. Several boxed bookshelves housed the material collected by the CCs – consisting of field management information and returned questionnaires. Each team was assigned a colour and each CC was designated a box identified by their team colour and their CD number which they used to deposit material for the TLs and DAs to process. Lockable filing cabinets were used to store the processed questionnaires. Questionnaires were marked off as being received at the PLO and were couriered to Adelaide for data entry.

### Staff training

The Community Engagement Officer (CEO), a local woman with extensive knowledge of the community, was hired on a part-time basis fourteen weeks before the census delivery started. The CS was hired eight weeks, and the TLs two weeks, before the census start date. The TLs were trained by the research fellows with the assistance of the CS. The training made extensive reference to the staff manual and FMS manual. These manuals were designed with simple step-by-step instructions and extensive use of graphics and diagrams. Manuals were given for pre-reading before the TLs and CCs began their formal training and employment. The scenarios to be role played in the training emphasised some of the more complex procedures outlined in the manuals. Some of the training scenarios included: how to introduce the study to the householder, what to do when three attempts to deliver or collect had failed to make any contact with the householder, what to do when a householder requested assistance or an interview, what to do when the householder refused, and what happened when you only collected some of the questionnaires from a household. It was emphasised during training that the accurate completion of the CCRB was a priority.

TLs had in-depth training, commencing two weeks prior to census delivery, and included:

1. Attending all the formal training sessions

2. Familiarising themselves with their districts – by driving around to view all the areas

3. Packaging the census material for each of their collectors

4. Scheduling CC visits to the PLO – every two days during the census period

5. familiarising themselves with the FMS

6. preparing the CC training.

CCs received four hours of training. Top-up CC training (one hour) occurred during the middle of the census period at the time of transition from delivery phase into collection phase and allowed opportunities for the CCs to share their stories and wisdom.

### Census promotion

**Figure 4:**
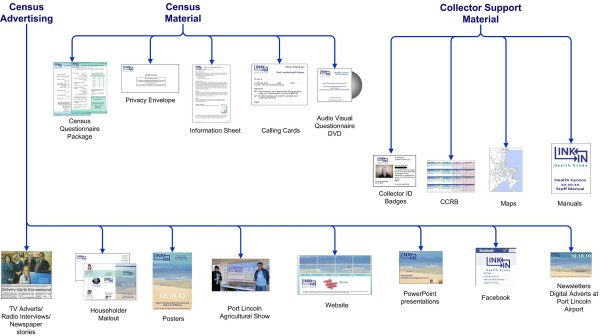
Health Census Resources.

A key component in the successful delivery of this census was the focus on community engagement and promotion carried out by the CEO and the one investigator who was based in Port Lincoln. The CEO had extensive links with the Port Lincoln community from a long career in local and state government. She carried out many of the promotional activities, for example, as guest speaker at community groups (Lions, Rotary, Mental Illness Fellowship, etc.) and scheduling marketing activities and media releases for live local radio and print media. The Port Lincoln investigator and CEO spoke to health organisations in both the private and public sector covering a broad range of health professionals. Once-off event marketing (e.g. local agricultural show) gave wide exposure and the repeated exposure in a range of media/meetings reinforced the upcoming health census. The message evolved throughout the 6 week census period for example, using both traditional and new media immediately before the census commenced, mid-way through, and at census closure (see Figure 4) [13] Additional file 2. Members of the local community also provided valuable endorsements which were used in the promotion of the census locally. The research team made a commitment to use local businesses where possible.

Documenting this community engagement work allowed creation of a database of local contacts, which has since been used in a Knowledge Translation strategy for dissemination of the census results to the Port Lincoln community and health service organisations.

### Census process results

An accurate household response rate could be calculated because the CCs identified and reported all the households in their CD. The household response rate was calculated, excluding invalid households (7%, N = 447), e.g. dwellings confirmed as derelict/empty for the 6 week census period and holiday homes. The valid household response rate for the health census was 75% (N = 4425). A total of 15% (N = 889) of households refused to participate in the census, 5% of households (n = 272) could not be contacted but appeared to be occupied, and another 5% (n = 306) were contacted at delivery but were unable to be contacted at collection or did not return data by pre-paid envelope. Collection success was greater when face-to-face contact was made at delivery compared to when the questionnaires were left in the letterbox at delivery (89% vs 64%). When face-to-face contact could neither be made at delivery nor collection, the questionnaire return rate fell to 26%.

Calculating individual response rate, as opposed to the household response rate, was problematic as the accurate number of individuals residing in a household was only ascertained by the CC when face-to-face contact was made. Consequently, if no contact was made the number of people living in the household was unknown. In this circumstance the CC left a census package in the letterbox which included four CIQs. Analysis of the FMS data indicated that on average two eligible people lived in a household, in each of the 26 CDs. Thus, for households where no contact was made, it was assumed that two eligible residents lived there. The estimate of individual response rate was 74% (N = 7956), which is consistent with the Australian Bureau of Statistics estimate of Port Lincoln adult population in 2010 of 10608.

Calculation of household response rates was deliberately conservative ensuring that the 75% household response rate and the 74% individual response rate were reliable figures. Our approach took into account the following:

1. We did not assume that households which could not be contacted were empty, but inferred an occupancy of 2 adults.

2. Where the number of questionnaires collected was less than the number delivered at face-to-face contact, the total delivered was deemed to accurately reflect the number of people living in the household.

3. Collected questionnaires were screened to ensure that duplicates were removed, a situation that occurred if a resident filled in the questionnaire multiple times.

4. CCs were trained to confirm the number of eligible residents in a household at both delivery and collection.

5. Quality control processes were systematically undertaken, including specific checks on undercount in each of the CDs by comparing a CC’s CCRB entries with counts taken by the TLs, CS and the research fellows by driving around the CDs and specific streets. These were particularly important in verifying the performance of a CC.

6. Quality checks were also done to ensure that householders had indeed been contacted or refused participation, depending on what the CC had recorded in the CCRB.

The CEO and Port Lincoln investigator’s promotion was observed to be useful, as evidenced by CCs reporting that overwhelmingly residents were aware of the LINKIN Health Study and specifically the 10.10.10 health census. Furthermore, the PLO had approximately 60 enquiries (phone, email and in-person visits) from residents wanting to ensure they had not been forgotten and they would be getting their census forms soon. Some of these enquiries were from people in neighbouring rural areas who wanted to know if they too could fill in the health census; some disappointment was detected when they were told that the boundaries defining the census denominator did not include their household.

## Discussion

The health census design as described in this paper differed from the mandatory Australian Government Census as we required multiple delivery and collection attempts with a specific focus on face-to-face contact rather than simply leaving the census material in a letterbox. We also specified that attempts had to be spaced apart and should include day time visits, evening visits and weekend visits to ensure maximum opportunity for a face-to-face contact. This more labour intensive approach gave an opportunity to explain the census, and to support participation for those participants known to be at home and resulted in a markedly improved response rates (89% participation when a face-to-face delivery was achieved compared to 26% participation when no contact was made with householders who may have been away or were not available for additional explanation).

Given equivalent resources, the LINKIN health census design could be easily adapted to a variety of regions in order to gain a crucial baseline population view for the purposes of needs analysis, evaluation, system change and infrastructure and policy development. The importance of gaining insights into the health needs and health service usage patterns of Aboriginal Australians was addressed through employing a different approach to data collection in neighbourhoods where there was a high proportion of Aboriginal households. This involved employment of an Aboriginal TL and CCs to emphasise culturally appropriate contact. This census method could also be adapted to account for language and literacy barriers by altering the mode of delivery to interview participation.

There were no major obstacles putting this method into practice, however, upon reflection the following improvements might be considered for future census implementation.

### Promotion suggestion

The designated census date was a useful promotional tool; however, it caused confusion for householders when face-to-face delivery was delayed and they had not received their forms by the 10.10.10 date. In practice the delivery and collection phases were blurred. To pre-empt community anxiety, promotion should stress that delivery of questionnaires is ongoing throughout census period.

### Personnel changes

• The management of the census would be better served by having a Field Manager and a Data Manager instead of the CS position. The Data Manager would be responsible for quality assurance data issues, would liaise with TLs on data quality and manage the heavy workload of DAs. This position would allow the FMS to be used to its full capacity. The Field Manager would focus on supervision of field based operations, and include liaising with TLs in the supervision and efficient data collection by CCs.

• Team leaders could manage an increased number of CCs (8–9).

• Pairing of CCs in a team should be considered to provide peer support on the ground and to even out workloads.

• Given that CCs were required to work independently, with irregular and out of office hours, careful consideration should be given to their contract arrangements. Clear communication of contract requirements, including occupational health and safety issues, should be explicitly stated at the outset.

• CCs should be relatively fit given the physical nature of their work.

### Census design tips

• To encourage maximum attempts by CCs to gain face-to-face contact with householders, delay distributing the reply paid envelopes to CCs until the end of the collection period.

• For efficient distribution of census questionnaires and related material, pass copies to CCs as they need it rather than in a single batch at the beginning of the census period.

• Secure and sturdy binding of CCRB is important for ease of use in the field and for photocopying purposes. While this may appear a minor matter, poor binding of the CCRB caused widespread frustration and difficulties using this document for CCs and TLs.

• Where possible, questionnaire data could be inputted on site to avoid shipping risks and costs associated with processing census material at another site (census data was couriered to the Data Management and Analysis Centre at the University of Adelaide).

### Staff training improvements

• Using a cascade model to training led to some inconsistent advice given to CCs (despite the provision of a detailed staff manual). Given the short timeline and the prescriptive nature of field processes, we would recommend that the research fellows conduct all training.

• Greater ongoing emphasis on FMS training especially for TLs, throughout the census period.

The LINKIN health census method demonstrates how it is possible to comprehensively gather person-based health data that is tied to social determinants of health, particularly from traditionally hard to reach populations and sub-populations. Most importantly, this method can collect data from sub-populations that may not be accessing health services, as well as sub-populations that access those health services where transactional data is not systematically recorded.

## Abbreviations

CC: Census collector; CCRB: Census collector’s record book; CD: Census district; CEO: Community engagement officer; CHQ: Census household questionnaire; CIQ: Census individual questionnaire; CS: Census supervisor; DA: Data administrator; FMS: Field management system; LGA: Local government area; PLO: Port Lincoln office; TL: Team leader.

## Competing interests

The authors declare that they have no competing interests.

## Authors’ contributions

CEH-L contributed to the design and implementation of the study, supervised the field activities and drafted the structure of the text. CEH-L, JN, AK, DW, AW, JK, JB and JB contributed to the overall design of the study, designed the study’s analytic strategy, reviewed and contributed to all sections of the text. All authors read and approved the final manuscript.

## Pre-publication history

The pre-publication history for this paper can be accessed here:

http://www.biomedcentral.com/1472-6963/12/321/prepub

## Supplementary Material

Additional file 1 **Table S1.** Equipment List for LINKIN Health Census, Port Lincoln, Australia, 2010. Click here for file

Additional file 2 **Documentation for permission to reference Figure **4**.** Click here for file
