# Supplementary material for: The LINKIN Health Census process: design and implementation
Source: BMC Health Serv Res. 2012 Sep 18;12:321. doi: 10.1186/1472-6963-12-321 (PMC3546420; doi:10.1186/1472-6963-12-321)
Supplement: Additional file 1 — Table S1. Equipment List for LINKIN Health Census, Port Lincoln, Australia, 2010. [file 1472-6963-12-321-S1.docx]

## Appendix 2

Equipment List for Linkin Health Census, Port Lincoln, Australia, 2010

| **Category/Item** | | **Quantity** | **Comment** |
| --- | --- | --- | --- |
| ***Printing Requirements for Census*** | | | |
|  | CCRB booklets | 35 | 1 for each CC with some reserves. Each booklet catered for 240 households |
|  | CCRB Top up booklets | 10 | For CDs with many households, provided space for another 100 households. Needed to print more in the field as many more households than previously identified in National Census held 4 years earlier |
|  | Individual Questionnaires | 13K | Needed to print another 1K in the field |
|  | Household Questionnaires Private | 5.5K | Needed to print another 200 in the field |
|  | Household Questionnaires Non-private | 100 |  |
|  | Information Sheets | 5.5K |  |
|  | Privacy Envelopes | 13K | Needed to print another 1K in the field |
|  | Calling Cards | 4K | 4 per A4 sheet |
|  | Census Staff Manual | 40 | For all Port Lincoln staff |
|  | FMS User Manual | 8 | For TL, DAs and CS only |
|  | Census District Maps | 27 maps x 3 | One copy was laminated for use in the field. Probably did not need the third copy in hindsight |
|  | CDs with Maps loaded | 35 | In hindsight probably not necessary |
|  | Reply-Paid Envelopes with Logo | 500 |  |
|  | Photo ID name badges | 30 | For each CC, generated on site in Port Lincoln |
| **Equipment for Census** | | | |
|  | Staplers for CCs | 30 | To staple together HIQ and CIQ |
|  | Satchels | 30 | For CCs to use in the field |
|  | Bandaids | 100 | A few were put in each satchel for CCs |
|  | Pencils + extra leads | 60 | For CCs |
|  | Clipboards | 30 | For CCs. These were covered with cheat sheet explaining outcome codes etc, and dot points about the introduction spiel |
|  | Lanyards | 30 | For CCs Photo IDs |
|  | Cardboard boxes | 30 | Recycled paper boxes labelled in the appropriate team colour and with the CD clearly marked on it. Used in the PLO to store field data and collected questionnaires. |
|  | Ring Folders | 30 | labelled in the appropriate team colour and with the CD clearly marked on it. Used in the PLO to store processed field data. |
|  | Manilla folders in 7 colours | 30 of each colour | Used to separate the various components that CCs used in the field, ie. CIQs, HIQs, Info Sheets, Calling cards, envelopes, etc |
|  | Clear plastic sleeves | 100 | For leaving questionnaire packages. In the end the CCs used these to arrange questionnaire packs for all householders and thus several thousand of these were later ordered |
|  | DVD AudioVisual version of the questionnaires | 30 | These were supplied for persons with literacy issues and assisted them in completing the questionnaires. |
|  | Hole puncher | 2 | Used by DAs when filing CCRB photocopies in the ring folders |
| **Census Infrastructure** | | | |
|  | Rooms | 3 | PLO for the TLs and DAs. Two additional offices for CS and the Community Engagement Officer |
|  | Computers | 6 desktop  3 laptops | Desktops in the main census room were leased for census period |
|  | Printer | 1 | BW for printing reports from FMS – leased for census period |
|  | Photocopier | 1 | Needs to be fast and robust |
|  | Backup drives | 2 | Data from the FMS was backed up daily onto one drive and removed from the PLO overnight |
|  | Telephones | 3 | 1 in each office |
|  |  |  |  |
| **Promotional material** | | | |
|  | Pamphlets | 7K | All part of the package mailed to all households in the Port Lincoln area. |
|  | Magnets | 6.5K |  |
|  | Mailout Envelopes with Logo | 6.2K |  |
|  | A3 posters | 120 |  |
|  | A4 posters | 20 |  |
|  | Banner and conflutes | 1 | Put up at various events around the city of Port Lincoln |
|  | Stamps and Edible Ink | 3 | Linkin Health Census Logo stamps used to brand free fruit at show and on various documents |
|  | Website |  | URL placed on all advertising, included dedicated Linkin Health Study email address for the public to contact research team |
|  | Dedicated phone line | 1 | Number placed on all advertising and was the community’s way of contacting research team |
|  | Facebook |  | As a way of reaching the adolescents and young adults |
|  | Scrolling LCD advertising | 1 | Situated at the Port Lincoln airport |
|  | Television advertising | Numerous | Advertising at key points, start, mid, end of census period |
