# Supplementary material for: The LINKIN Health Census process: design and implementation
Source: BMC Health Serv Res. 2012 Sep 18;12:321. doi: 10.1186/1472-6963-12-321 (PMC3546420; doi:10.1186/1472-6963-12-321)
Supplement: Additional file 2 — Documentation for permission to reference Figure 4. [file 1472-6963-12-321-S2.pdf]

From: Dawn Hunter
To: Elizabeth Hoon
Subject: RE: Poster for online display
Date: Tuesday, 18 October 2011 9:43:44 PM
Attachments: image001.png
image002.png

Dear Elizabeth

Thank you for sending in your poster. I have sent this to my colleague to put online. In terms of using the table, that would be fine-as long as you reference.

Kind regards
Dawn

Dawn Hunter
Acting Managing Editor
Royal Society for Public Health
John Snow House
59 Mansell Street
London
E1 8AN
www.rsph.org.uk

DD: +44 (0) 20 7265 7332
Switchboard: +44 (0) 20 7265 7300
Fax: +44 (0) 20 7265 7301

RSPH has moved offices!
Please note our new address and telephone numbers above.

From: Elizabeth Hoon [mailto:elizabeth.hoon@adelaide.edu.au]
Sent: 18 October 2011 02:41
To: Dawn Hunter
Subject: Poster for online display

Dear Dawn
Here is the poster for online publication.

I would like to seek authorisation from RSPH to use one of the tables from this poster in another publication- the table is titled 'Census Resources'. I would of course cite RSPH conference proceedings as the original source.
I look forward to hearing from you

Regards
Elizabeth

LINK IN
Health Study

Elizabeth Hoon
Research Fellow & Project Co-ordinator
Linkin Health Study
School of Populaton Health and Clinical Practice
The University of Adelaide
122 Frome Street
Adelaide 5005
ph: +61 (0)8 8313 1567
Email: elizabeth.hoon@adelaide.edu.au
website: www.linkinhealth.org.au
facebook: www.facebook.com/LinkinHealthStudy

CRICOS Provider Number 00123M

This email message is intended only for the addressee(s) and contains information which may be confidential and/or copyright. If you are not the intended recipient please do not read, save, forward, disclose, or copy the contents of this email. If this email has been sent to you in error, please notify the sender by reply email and delete this email and any copies or links to this email completely and immediately from your system. No representation is made that this email is free of viruses. Virus scanning is recommended and is the responsibility of the recipient.
